# The first-to-test bias: Impact of testing order on assigning responsibility for contagion

**DOI:** 10.1371/journal.pone.0297965

**Published:** 2024-03-14

**Authors:** Julian Givi, M. Paula Fitzgerald

**Affiliations:** Department of Marketing, John Chambers College of Business and Economics, West Virginia University, Morgantown, West Virginia, United States of America; University of Haifa, ISRAEL

## Abstract

When a contagious disease spreads, people wonder about who to blame for transmission. Herein, we document a novel bias, the “First-To-Test” bias, that emerges when individuals assign responsibility for contagion within a dyad. People tend to believe that the member of the dyad who tested positive first is more likely to have given the disease to the other member, even when all other relevant factors are held constant. That is, while using testing order as a basis for assigning responsibility for a dyad’s contraction of a contagious disease may be rational in cases where all other relevant factors are not held constant, we show that individuals are more likely to allocate responsibility to whoever tested positive first even when these relevant factors are held constant. This overgeneralization bias emerges regardless of whether the evaluator is an outside observer or the member of the dyad who tested positive first. While we explore this bias with COVID-19 and strep throat, it has implications for other contagious diseases such as sexually transmitted infections (STIs) and illnesses often spread among school children (e.g., influenza, whooping cough). We conclude by discussing its implications for patients and organizations.

## Introduction

When it comes to the spread of contagious diseases, a common question that gets asked is, “How did this happen?” As the disease passes among friends, partners, family, coworkers, and so on, it is natural for those who contract it, as well as others, to wonder about the order the disease spread among those who acquired it. Indeed, in a pilot study, we found that 80% of people have (vs. have not) wondered about the order of transmission in cases where a dyad contracts a disease. Moreover, these thoughts about contagion can sometimes manifest in more serious, visceral forms, such as when individuals struggle with the guilt stemming from the thought that they may have passed a disease to a loved one who was killed by it [1] or look to blame others upon contracting the disease themselves [2].

Beyond merely satisfying curiosity, understanding the order of disease transmission is critical to many aspects of public health, including contact tracing, understanding the impact of super spreader events and the dynamics of the spread of a disease over time, implementing interventions to control or mitigate the impact of a disease, determining how to distribute vaccines and other resources, and health messaging.

Recently, much has been written about one contagious disease, COVID-19. Important work in many journals focused on the intersection of COVID-19 and psychology has been published [3, 4]. One segment of this literature studies the psychology involved with assigning responsibility for the spread of COVID-19 and other infectious diseases. Work in this area generally takes a broader view of responsibility attribution, such as by studying how Asian individuals and Asian-owned firms were treated because of the notion that COVID-19 originated in China [5], and illuminating how people fail to consider systematic inequalities (e.g., type of employment, access to green space, housing status) when blaming others for catching an infectious disease [6]. As another example, research has documented how some people assign responsibility to the rich—who can afford to travel—for spreading diseases [7].

In the present work, we build on this line of research by studying the psychology involved with assigning responsibility for the spread of infectious diseases at a more finite level, specifically, the dyadic level. This is a level of examination that has received minimal, if any, attention. We demonstrate that people attributing responsibility for the spread of a contagious disease within a dyad exhibit a systematic bias whereby they tend to believe that, holding all else constant (e.g., symptom onset, symptom severity, etc.), the member of the dyad who tested positive first is more likely to have transmitted the disease to the other member, than vice-versa. While assigning responsibility based on testing order may be rational in cases where other relevant factors are not held constant, we study cases in which relevant factors are, indeed, held constant. In other words, we show that if two people have acquired the symptoms of a contagious disease at the same time, share the same vaccination status, have the same histories of social interactions with others, and so on, but one of them was tested before the other, the person who was tested first will be seen (by outside parties, themselves, etc.) as more likely to have been responsible for passing the contagious disease to the person who was tested second (than vice-versa). We call this phenomenon the “First-To-Test” bias.

Our research contributes to the literature on the psychology involved with how people assign responsibility for the spread of infectious diseases by providing an initial foray into perceptions about dyadic-level responsibility and contagion. Beyond this literature, our work connects to the wide body of research on attribution and anchoring theory, as well as research on overgeneralization and relevance insensitivity, as we will discuss. Moreover, our research has practical implications for patients and organizations alike, as we delineate in detail in the General Discussion. In what follows, we first discuss related literature that provides insight into the cause of the First-To-Test bias. Next, we present three studies that document the First-To-Test bias. We then conclude with a discussion of the implications of this work.

## The cause of the first-to-test bias

Whenever the members of a dyad contract a contagious disease, multiple factors can be relevant to determining which member was more likely to have spread it to the other. For example, holding all else constant, the timing of symptom onset, the frequency and duration of social activities with others, vaccination status, and the amount of time since the dyad last saw each other, are all relevant.

That said, holding all else constant, one factor that is *not* relevant to determining which member of a dyad was responsible for transmitting the disease to the other is the order in which the two parties tested positive. In other words, holding constant the timing of symptom onset, social activities with others, vaccination status, and a myriad of other aspects, if one member of the dyad tested positive prior to the other, this factor is irrelevant to assessing which member passed the disease to the other. In the present work, we show that people fail to realize this. They believe that, holding all else constant, the member of the dyad who tested positive first is more likely to have transmitted the disease to the other member, than vice-versa. As mentioned, we call this the First-To-Test bias.

We suggest that the First-To-Test bias can be explained, in part, by considering attribution and anchoring theory [8–10], along with relevance insensitivity and overgeneralization [11, 12]. Specifically, when all relevant factors are held constant and only testing order differs, the covariation model of attribution suggests that a person will hone in on the aspect of the situation that is distinctive and covaries with the actions leading up the dyad experiencing the disease [10]. In this case, the distinctive action is the first person going and obtaining the positive test result—everything else is held constant. Individuals may then rely (i.e., anchor) on the lay belief (and general scientific premise) that causes precede effects. Thus, people attempting to determine which member of a dyad transmitted a contagious disease to the other may initially anchor on the idea that the member who tested positive first was the transmitter, an inference consistent with individuals’ general beliefs about cause preceding effect. This anchor results not only because of the belief that causes precede effects, but also because, when other relevant factors are *not* held constant, it can be rational to think that the member who tested positive first was the source, leading to overgeneralization. For example, if one member of a dyad developed symptoms a few days before the other and thus got tested first, it would be logical to think that they were the transmitter. As another example, if both members developed symptoms simultaneously, but one member recently engaged in risky behaviors (e.g., attended events with large numbers of people during a pandemic, attended school classes during an influenza outbreak, engaged in unprotected sex) and thus got tested first, it would be rational to think that this person acquired the disease first. Because there are many cases where it is logical to believe that the member of a dyad who tested positive first was the transmitter, people may naturally overgeneralize and see testing order as relevant even when it is not and anchor on that general belief when making an attribution. In other words, individuals may focus on the notion that that the member who tested positive first was the transmitter, even when testing order is irrelevant. This is consistent with recent research on relevance insensitivity, in which people overgeneralize the importance of factors that are sometimes relevant to situations in which they are not at all relevant [11, 12].

Systematic biases from anchoring and overgeneralization effects are common in social psychology [13]. These anchors are overweighed, usually without the person’s knowledge, in judgments [14]. Indeed, much evidence across a wide array of settings shows that it is challenging to sufficiently adjust away from anchors [8, 9], suggesting that insufficient adjustment may occur in the present context as well. Thus, upon overgeneralization leading one to form the anchor that the member of the dyad who tested first was the transmitter, a person is unlikely to then sufficiently adjust their belief to account for the fact that none of the factors actually relevant differed across the two members of the dyad. That is, they may bias their assessment of the causal order of transmission by relying on the belief that whoever tested positive first for the disease is the most likely to have spread it. In sum, there is good reason to suspect that the First-To-Test bias may arise because of anchoring (due to overgeneralization) and insufficient adjustment.

## The present research

We next present three studies that document the First-To-Test bias, using multiple diseases across studies. In each study, we report all manipulations and measures, include all participants who passed the attention checks in the analyses, and chose the sample size prior to beginning data collection. The West Virginia University Office of Human Protections approved of this research. Participants provided written consent in all studies. Our data and materials can be accessed here: https://osf.io/4hp5a/?view_only=cf90f69df0f84a0b9dcfa2571f42ec89.

## Study 1

Study 1 served as an initial demonstration of the First-To-Test bias. Participants read a vignette in which two people spent time together and then developed similar COVID-19 symptoms simultaneously. One person then tested positive for COVID-19 and informed the other person. The other person then tested positive for COVID-19 as well. After reading the vignette, participants indicated which person they believed gave COVID-19 to the other.

### Method

One hundred ninety-six Prolific participants (195 after exclusions: 55% female, 43% male, 2% non-binary, 1% preferred not to respond; *M*_Age_ = 39.2, *SD*_Age_ = 13.3) completed the study, which was run in full on 5/25/2023. Participants were first provided with the following information about two friends, Alex and Jordan: they are the same gender; they are about the same age and of similar overall health; they both received the first two COVID-19 vaccines but neither take additional preventive measures to avoid spreading COVID-19 (e.g., wear masks, socially distance, receive vaccine boosters); and they both have COVID-19 tests available to them at a nearby, well-regarded hospital. This information was provided to keep everything (besides testing order, see below) constant across the two friends.

Next, participants read a vignette in which the two friends watched a movie at a movie theatre on a Friday night. They sat next to each other but not very close to others. The following Monday, both woke up experiencing similar COVID-19 symptoms (e.g., fatigue, sore throat, etc.) of similar severity at their respective homes. One of them (counterbalanced) then got tested for COVID-19 at a nearby, well-regarded hospital. The test came back positive, so the person who got tested sent a text to the other person that informed them of the news. The other person then got tested at the same hospital, and their test also came back positive.

After reading the vignette, participants chose which person—the one who tested positive first, or the one who tested positive second—they believed gave COVID-19 to the other person. Next, all participant responded to a secondary measure of their belief regarding which person gave COVID-19 to the other person, but we varied between-subjects whether this measure used a six-point scale (1 = More likely that the person who tested positive second gave it to the other person, 6 = More likely that the person who tested positive first gave it to the other person) or a seven-point scale (1 = More likely that the person who tested positive second gave it to the other person, 7 = More likely that the person who tested positive first gave it to the other person). Varying the scales in this way allowed us to test whether responses become less severe when there is a neutral option available for participants to choose, like with a seven-point scale. Moreover, binary choice dependent measures and continuous dependent measures each offer unique advantages and disadvantages [15], making it important to employ both.

Participants then responded to an attention check and a series of demographic and background questions. Regarding the former, they selected what the survey was about (COVID-19) from a list of four options. Regarding the latter, they reported their age, gender, political affiliation, attitude toward COVID-19 (1 = It is not a serious issue, 7 = It is a very serious issue), and attitude toward COVID-19 testing (1 = Testing is a waste of time, 7 = Testing is very important). Across studies, our results were generally consistent across all the demographic and background questions that we asked, so we do not report any analyses with them.

### Results and discussion

**Fig 1:**
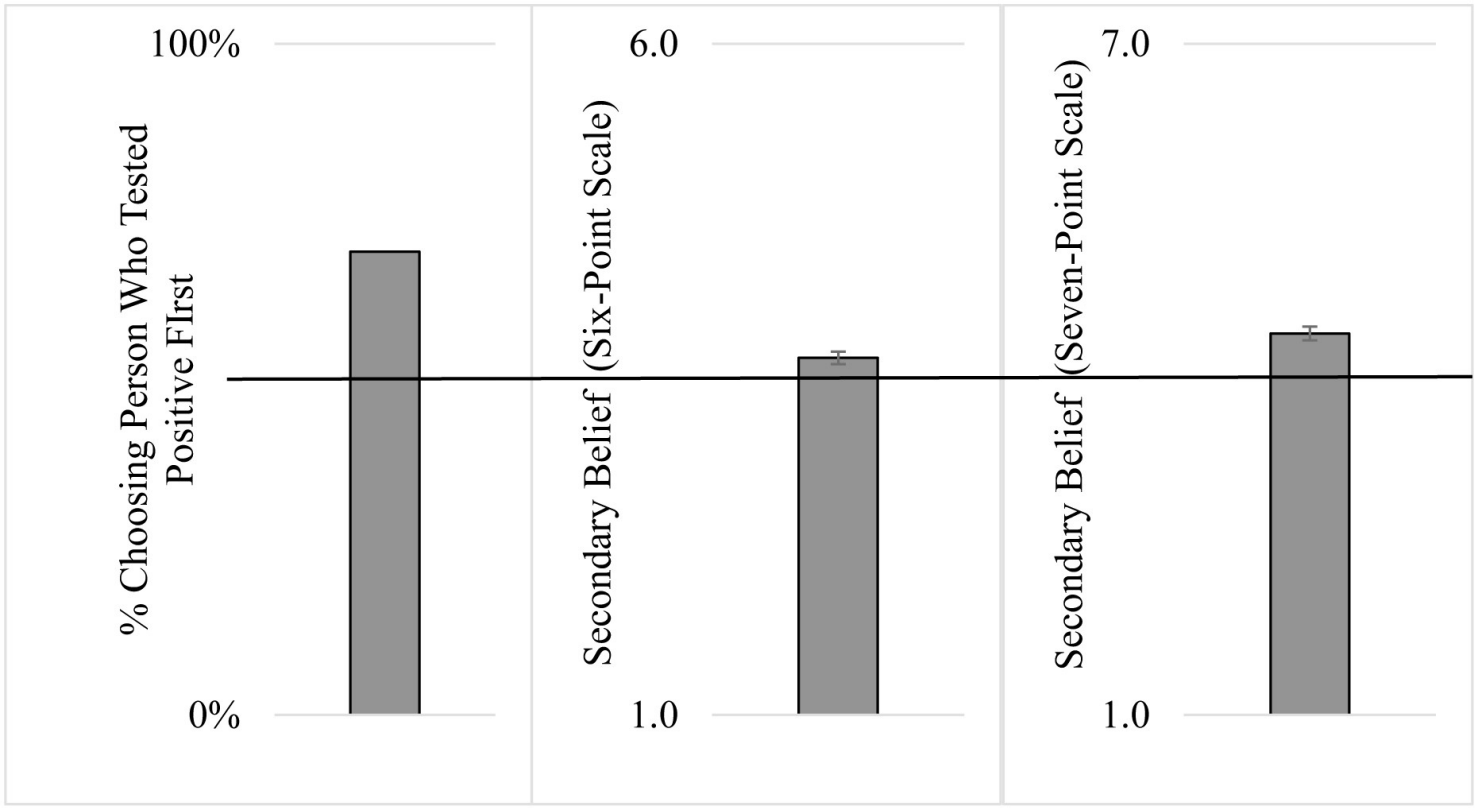
Study 1 results. Error bars in the second two panels represent standard error of the mean. Solid line across the middle of the graphs represents comparison value (i.e., 50%, 3.5, and 4.0, respectively).

Even though all was held constant across the two friends except for testing order, participants believed that the person who tested positive first was responsible for spreading COVID-19 to the person who tested positive second (see Fig 1). Specifically, 69% of participants indicated that the person who tested positive first gave COVID-19 to the person who tested positive second (*p* < .001 in a binomial test against 50%, *g* = .19). The secondary belief measures mirrored this result. Participants who responded on a six-point scale had a mean response above the middle of the scale (3.5 vs. *M* = 3.66, *SD* = .91; *t*(97) = 1.78, *p* = .078, *d* = .18), as did those who responded on a seven-point scale (4 vs. *M* = 4.41, *SD* = 1.20; *t*(96) = 3.39, *p* = .001, *d* = .35).

These results provide an initial demonstration of the First-To-Test bias. Moreover, we ran a post-test to ensure the study was believable, thereby improving ecological validity. Participants were presented with the Study 1 scenario and then rated its believability (1 = Not at all, 7 = Very). The scenario was viewed as highly believable (*M* = 6.63, *SD* = .70; one-sample *t*-test against the midpoint: *t*(47) = 25.86, *p* < .001, *d* = .70). We ran another post-test to ensure the study did not provide too much detail. Participants were presented with the Study 1 scenario and then indicated whether it provided too much detail (Yes vs. No). Only 13% of participants said it did (*p* < .001 in a binomial test against 50%).

## Study 2

Study 2 looked to again demonstrate the First-To-Test bias while extending it to a new type of disease: strep throat. To do so, Study 2 employed a similar procedure as Study 1, except that the disease involved was strep throat rather than COVID-19. Finding evidence for the First-To-Test Bias with a new type of disease would expand the generalizability of our work.

### Method

103 Prolific participants (102 after exclusions: 48% female, 47% male, 5% non-binary; *M*_Age_ = 35.1, *SD*_Age_ = 10.3) completed the study, which was run in full on 5/30/2023. The main portion of the study was similar to Study 1, except that the relevant disease was strep throat, rather than COVID-19. Participants learned information about two friends (again provided to keep everything constant across the two friends), read a scenario involving the two friends testing positive for strep throat after spending time with each other, and indicated which friend they thought gave strep throat to the other. Participants then responded to an attention check and a series of demographic and background questions. Regarding the former, they selected what the survey was about (strep throat) from a list of four options. Regarding the latter, they reported their age and gender.

### Results and discussion

Even though all was held constant across the two friends except for testing order, participants believed that the person who tested positive first was responsible for spreading strep throat to the person who tested positive second. Specifically, 71% of participants indicated that the person who tested positive first gave strep throat to the person who tested positive second (*p* < .001, *g* = .21). These results provide another demonstration of the First-To-Test bias and expands the generalizability of our work.

## Study 3

Whereas Studies 1 and 2 tested whether the First-To-Test bias emerges when outsiders attempt to determine which member of a dyad transmitted a disease to the other, Study 3 examined whether it arises when the person making the assessment is a member of the dyad themselves. Finding evidence for the First-To-Test Bias when an individual is personally involved in the situation would expand the practical implications of our work.

### Method

Two hundred eighty-five Amazon Mechanical Turk (MTurk) participants (284 after exclusions: 45% female, 54% male, < 1% non-binary, 1% preferred not to respond; *M*_Age_ = 41.5, *SD*_Age_ = 11.8) completed the study, which was run in full on 5/31/2022. Participants were assigned to one of two between-subjects conditions (Initial Tester: *Other* vs. *Friend*). They began by providing their vaccination status (unvaccinated, partially vaccinated, fully vaccinated, fully vaccinated and boosted, prefer not to respond [those who selected this option skipped the remainder of the study and thus did not provide data for the analyses]) and listing a friend with the same vaccination status. Participants then read a vignette in which they and their friend attended a movie on a Friday night and woke up the following Monday experiencing similar COVID-19 symptoms. One of the two then got tested. Which person this was—the friend or the participant—varied based on condition. In the *Initial Tester–Other* condition, it was the friend, while in the *Initial Tester–Self* condition, it was the participant. The test came back positive, so the person who got tested sent a text to the other person that informed them of the news. The other person then got tested, and their test also came back positive.

After reading the vignette, participants chose which person—the one who tested positive first, or the one who tested positive second—they believed gave COVID-19 to the other person. Next, they responded to a secondary measure of their belief regarding which person gave COVID-19 to the other person, using a seven-point scale (1 = More likely that the person who tested positive second gave it to the other person, 7 = More likely that the person who tested positive first gave it to the other person). Participants then responded to the same attention check and series of demographic and background questions from Study 1.

### Results and discussion

Even though the two people experienced similar symptoms that started at the same time and had the same vaccination status, participants in both conditions believed that the person who tested positive first was responsible for spreading COVID-19 to the person who tested positive second. Specifically, in the *Initial Tester–Other* condition, 64% of participants indicated that the person who tested positive first gave COVID-19 to the person who tested positive second (*p* < .001, *g* = .14), while in the in the *Initial Tester–Self* condition, 60% of participants indicated that the person who tested positive first gave COVID-19 to the person who tested positive second (*p* = .015, *g* = .10). These percentages did not differ (Wald χ^2^ (1, *N* = 284) = .45, *p* = .501, φ = .04). The secondary belief measure mirrored these results. In the *Initial Tester–Other* condition, participants had a mean response above the middle of the scale (4 vs. *M* = 4.41, *SD* = 1.26; *t*(139) = 3.90, *p* < .001, *d* = .33). The same was true for participants in the *Initial Tester–Self* condition scale (4 vs. *M* = 4.19, *SD* = 1.33; *t*(143) = 1.76, *p* = .081, *d* = .15). These means did not differ (*t*(282) = 1.43, *p* = .154, *d* = .17). In sum, these results demonstrate that the F2T bias emerges even when someone is part of the dyad that has acquired COVID-19, regardless of whether they or the other member of the dyad tested positive first.

## General discussion

Across three studies, we demonstrated a novel bias that colors people’s assessments of who is responsible for the spread of diseases within dyads: the First-To-Test bias. Specifically, we showed that, holding all else constant, people believe that the member of the dyad who tested positive first is more likely to have transmitted the disease to the other member, than vice-versa. Moreover, we demonstrated that this bias emerges regardless of whether someone is part of the dyad themselves or an outside observer.

Studying the medical facets of COVID-19 and other diseases [16] is obviously critical, but so too is studying the psychology involved with diseases, which is why there have been several papers on this topic [3, 4]. In regard to the present research, this work is important largely because it provides an initial foray into perceptions about responsibility assignment for disease contagion at a dyadic level, thus adding to extant literature which has studied this kind of responsibility attribution at broader levels [5–7]. Our work is also important because it is the first to apply anchoring and adjustment, along with overgeneralization and relevance insensitivity, to disease contagion perceptions. Moreover, there is a visceral side to beliefs regarding the spread of infectious diseases. In an additional study (see S1 File), we found that when someone tests positive for an infectious disease first, the First-To-Test bias spurs negative emotions such as shame and guilt, and feelings like blame and at fault. Conversely, when someone believes that another person gave a disease to them, they may harbor feelings of ill will, and the relationship between the two parties can suffer [2]. Additionally, even when someone has not contracted a disease themselves, they may still hold negative feelings toward those who they feel are responsible for spreading the disease. This may be in part because of a belief in a just world, whereby individuals feel that others get what they deserve. In other words, if someone contracted a disease, it is simply because they were not careful enough [17]. At the extreme, this can lead to the physical and emotional abuse of others, as was observed rather often early in the pandemic when people of Asian descent were harassed, abused, and even murdered because of COVID-19’s assumed origin [18].

Our work is also important and relevant to marketing practitioners in the healthcare and public health domains as well as to policy makers. We know from prior research that attribution errors—such as First-To-Test bias—lead people to stigmatize others, which is an important factor for policy makers to consider as they implement measures intended to limit the spread of diseases [19]. Also, testing for infectious diseases is key to limiting their spread. Accordingly, practitioners in these domains regularly release marketing messages intended to encourage patients to get tested for infectious diseases. Yet, our results suggest that patients may be hesitant to get tested because by being the first one within a dyad or friend group to do so, they will, consequently, be the one who is blamed and who will experience negative emotions such as guilt and shame. Marketers may be able to quell this concern by ensuring patients in advertisements that, just because they get tested, they don’t deserve the blame. While refraining from testing is certainly a negative outcome, the First-To-Test bias likely has some positive ones as well. For example, if the First-To-Test bias leads a patient to believe they were responsible for a dyad contracting a disease, they may be more likely to inform others with whom they recently interacted about their positive test (so those other people can get tested as well). Moreover, feeling responsible in this manner may also prompt patients to be more likely to get tested for infectious diseases in the future.

Our findings also open multiple doors for future work. For example, there may be moderators of our findings that future research could explore, including potential cultural differences [20] and individual differences in information processing and reasoning [21]. Empirically examining downstream consequences, such as through the use of the behavioral guidelines for COVID-19 scale [3], would also be valuable. Also, while our work used COVID-19 and strep throat as contexts, the First-To-Test bias may apply to other diseases, such as monkeypox, influenza, and whooping cough. Sexually transmitted infections may also evoke the First-To-Test bias. According to the Mayo Clinic, individuals infected with an STI may or may not have symptoms (e.g., when infected with chlamydia or gonorrhea) or may experience symptoms intermittently (e.g., when infected with genital herpes; [22]). STIs are an understudied aspect of well-being, but according to the U.S. Centers for Disease Control and Prevention, reported cases of STIs are increasing (2.5 million cumulative cases of chlamydia, gonorrhea, and syphilis reported in 2021 [23]). HIV is another STI that could induce the First-To-Test bias, given people’s tendency to have biased perceptions when making contagion-related assessments for HIV [24, 25] (this is above and beyond the difficulties of making such assessments with medical methods [26]). Future research could and should test whether our findings hold for other diseases.

In addition, our results indicate that patients not only face the unpleasant possibility of finding out they have a disease when they choose to purchase and use a medical test (be it at home or in a healthcare facility). Patients, if they are the first in a dyad to take that responsible action, also risk taking the blame for spreading the disease, even if they are not “patient zero.” Identifying methods to minimize this bias would be a useful endeavor for researchers hoping to increase medical testing as a method of improving public health. Also, there are certainly limitations to using hypothetical studies as we did, so extending the present work by testing whether the First-To-Test bias emerges in the “field” would be a valuable endeavor. Finally, we explored cases in which all other relevant factors are known and held constant. We believe cases like these are not uncommon, such as when the dyad involves close friends, roommates, significant others, or family members. Here, each member of the dyad knows all the relevant information about the other person, and many of the factors are the same across the dyad (e.g., a married couple likely shares the same vaccination status). However, as mentioned earlier, in cases when other relevant factors are unknown or are not held constant, it may be rational to use testing order as a basis for assigning responsibility. For example, if one person gets symptoms before another, and thus the former gets tested first, then using testing order could be a rational course of action. Interestingly, however, there are also cases in which using testing order as a basis would be clearly suboptimal. For example, if one person gets symptoms before another, but the person who developed symptoms second gets tested first, it may be suboptimal to think this individual (who tested first) gave the disease to the other person. Future work could explore cases like these in which the First-To-Test bias may produce clearly suboptimal ways of thinking.

## Supporting information

S1 File (DOCX)
