# Supplementary material for: The first-to-test bias: Impact of testing order on assigning responsibility for contagion
Source: PLoS One. 2024 Mar 14;19(3):e0297965. doi: 10.1371/journal.pone.0297965 (PMC10939239; doi:10.1371/journal.pone.0297965)
Supplement: S1 File — (DOCX) [file pone.0297965.s001.docx]

**Supporting information**

**Method**

97 Prolific participants (no exclusions: 69% female, 31% male; *M*_Age_ = 36.6, *SD*_Age_ = 12.5) completed the study, which was run in full on 6/13/2023. The main portion of the study was similar to Study 1, except that participants imagined they were personally involved in the scenario as the person who tested positive first, and we used multiple, visceral dependent variables. Participants first thought about the friend who was closest to themselves who shared as many of the following characteristics with them as possible: gender, age, overall health, vaccination status, behavior in terms of taking (or not taking) additional preventive measures to limit the spread of COVID-19, and had access to COVID-19 tests at a nearby medical provider. They then read the scenario from Study 1, except that they and their friend were the people involved, and they themselves tested positive first. As our dependent measures, we asked participants which person (them, who tested positive first, or their friend, who tested positive second) deserved more blame, was more at fault, should feel more guilty, and should feel more shameful. Participants then responded to the same attention check and series of demographic and background questions from Study 1 (along with indicating their COVID-19 vaccination status).

**Results and discussion**

Even though all was held constant across the participant and their friend except for the fact that the participant tested positive first, participants believed that they were responsible for spreading COVID-19 to their friend. Specifically, 94% of participants indicated that they deserved more blame (*p* < .001, *g* = .44), 92% indicated that they were more at fault (*p* < .001, *g* = .42), 92% indicated that they should feel more guilty (*p* < .001, *g* = .42), and 93% indicated that they should feel more shameful (*p* < .001, *g* = .43).
